# Collectanea Medica

**Published:** 1813-04

**Authors:** 


### COLLECTANEA MEDICA,

CONSISTING OF

### ANECDOTES, FACTS, EXTRACTS, ILLUSTRATIONS, QUERIES, SUGGESTIONS, &c.

RELATING TO THE

*History or the Art of Medicine, and the Auxiliary Sciences.*

THE *Psylli* of the ancient world, of Egypt, Rome, &c. and the serpent-charmers of the modern, of India, America, and Abyssinia, are too well known by remaining records, or by the statement of modern travellers, to leave any doubt of there being something extraordinary in the fact itself, either as a real natural operation, or as some dexterous juggle.

The following account, as being of a more specific nature than most others, may possibly deserve some further inquiry.

"Don Pedro d'Orbeis y Vangas, a native of Santa Fé, in the year 1788, being at Margarita, met with a slave who possessed the power of charming the most venomous of the American serpents. After the negro had exhibited his skill, he was induced by a reward to promise a discovery of his secret. The next morning he returned with the leaves of a plant, called vejuco du guaco; and having bruised them, in the presence of Don Pedro, gave him two large spoonfuls of the juice to drink; then making three incisions between the fingers of each hand, he inoculated the Spaniard with the same juice, and performed a similar operation on each foot, and on each side of the breast; after which he informed him, that he was no longer accessible to the poison of serpents. Don Pedro then, after making the negro answerable for any ill consequences, took into his hands several times one of the serpents that had been brought by the slave the day before, without receiving the smallest injury from the animal. Encouraged by this first attempt, two domestics, being in like manner prepared by the guaco juice, went into the fields, and soon returned with another kind of serpent, equally venomous with the former, without sustaining any hurt. Another person being similarly prepared, and afterwards bitten by a poisonous serpent, received no further injury than a slight local inflammation. Since this period, Don Pedro has repeatedly caught serpents with his own hands, with absolute impunity, employing no further preparation than merely drinking a little of the guaco juice. The plant, whose effects are thus attested, has not as yet been admitted into any botanical system, but is amply described in a memoir by the Spanish gentleman already mentioned, inserted in a weekly paper published at Santa Fé. It is of the compound-flowered or syngenesious class. The stamina are five in number, united by their anthers into a cylinder, through which rises the pistil, with a deeply-divided summit. The corolla is monopetalous, infundibiliform, with five indentations; and of a yellow colour; each calyx contains four florets, and several of these grow together, forming a corymbus: the seeds are broad and feathered; the root is fibrous, perennial; the stem straight, cylindrical when young, but, when old, becomes pentagonal: leaves are heart-shaped, opposite, of a dark green mixed with violet, velvetty on the upper surface. It grows by the sides of rivulets, and in shady places, in the viceroyalty of Santa Fé."

It has been suggested that the *Vejuça du Guaço* may be a species of *Eupatorium*. In the form of its leaf, however, it does not agree with the *Eupatorium ayapana*, which grows in New Mexico, and which is there considered to be an antidote against the poisonous bites of serpents. The celebrated botanist MUTIS, who was living very lately at *Santa Fé*, could possibly explain this. It can hardly be admitted that so striking a fact as is above related, could have happened in 1788, if properly authenticated, without yet being more fully known.

### *Medical Reform.*

Before the time of Henry VIII. the different branches of the medical profession were exercised indiscriminately, and the pen and the pestle, the scalpel and the razor, were wielded by the same hand. That humane monarch, mindful of the "grievous hurt, damage, and destruction," which was inflicted upon his liege people, by the multitude of ignorant artificers, smiths, weavers, women, and magicians, who in those days practised the "science and cunning of physic and surgery," graciously enacted, that no person should exercise the profession in any part of his dominions, unless he was previously examined, approved, and admitted, by the bishop of the diocese in which he resided.* The same king subsequently instituted the royal foundation of the College of Physicians; † and the rights and privileges of that body were ratified and confirmed to them by various acts of parliament during his reign. Their powers and privileges were enlarged by acts of Charles II. James, Mary, and Elizabeth; the different branches of the profession were separated from each other, their liberties ascertained and determined, and powers given to the College of Physicians, which to this day they have the right to enforce. By these statutes they were made a body corporate, allowed to elect a president, to have a common seal, to purchase land, and to sue and to be sued. It was also enacted, that no person should practise physic in London, or within seven miles thereof, unless he be admitted a member of their body, nor in any part of England, unless he had been examined by the president and three of the elects of the college, except he be a graduate of Oxford or Cambridge, which "accomplished all things for his fourme without any grace." ‡ They were also discharged from keeping watch or ward, from being chosen constables, churchwardens, jurymen, and scavengers, and from other offices tending to "their great fatigation and unquieting, and to the peril of their patients, by reason they

* 3 Hen. VIII. c. 2. † 14, 15 Hen. VIII. c. 5. ‡ Ibid.

cannot cannot be sufficiently attended." * They were allowed to fine and imprison all persons violating their laws; to dissect yearly the bodies of six felons; to enter the shops of apothecaries and to hunt for bad drugs. They were endowed with an inquisitorial power over the other branches of the profession, the administration of which was amenable to their jurisdiction.

Henry VIII. enacted, that surgeons should be ordained by the bishop of the diocese, in the same manner as physicians. † Edward IV. had incorporated the barbers and the surgeons into one company; but it appears that in the time of Henry VIII. there was still a company of persons exercising the "mystery and craft" of surgery, who did not at the same time practise the "feats and crafts of barbery and shaving." ‡ These two companies of surgeons and barber-surgeons were therefore united, "to the intent that by their union, and often assembly together, the good and due order, exercise, and knowledge, in the said science and faculty of surgery, should be as well in speculation as in practice; and by their learning and diligent and ripe informations more perfect, speedy, and effectual remedy should be, than it hath been, or should be, if the said two companies of barbers and surgeons should continue severed asunder, and not joined together, as they before this time have been, and used themselves not meddling together." || Having united the surgeons and barber-surgeons, the same monarch separated the practice of barbery from that of surgery, and ordained that no person using any barbery or shaving "should occupy any surgery, letting of blood, or any other thing belonging to surgery (drawing of teeth onely except)," because in those days "persons using of the mystery or faculty of surgery, oftentimes meddle and take into their cure and houses such sick and diseased persons as have been infected with the pestilence, great pocks, and such other contagious infirmities, do use or exercise barbery, as washing or shaving, and other feats thereunto belonging, which is very perilous for infecting the king's liege people, resorting to their shops and houses, there being washed or shaven." § Those of the company who practised surgery were in like manner interdicted from the feats of barbery, and were compelled to place a sign at their doors, and to be freemen of the city of London. The members of the company were exempt from bearing arms, from being on watches and inquests; they were allowed to choose masters and wardens, to dissect yearly

* 32 Hen. VIII. c. 40. † 3 Hen. VIII. c. 2.

‡ 32 Hen. VIII. c. 42. || 32 Hen. VIII. c. 42. § Ibid.

four four bodies, and it was graciously made lawful for them to become the valets and servants of any of the king's subjects who were not barbers or surgeons. Physicians were allowed to practise surgery, but surgeons in no wise to exercise physic. Although surgeons were prohibited from practising physic, it was nevertheless ordained by act of parliament, that "divers honest persons, as well men as women, whom God hath endued with a knowledge of the nature, kind, and operation, of certain herbs, roots, and waters, to customable diseases," should be allowed to use and minister the same, according to their "cunning, experience, and knowledge;" * so that in fact this branch of the profession was rendered subservient to every other, a circumstance which was probably rendered necessary by the conduct of the surgeons, whom we find represented as in those days "minding onely their own lucres, and nothing the profit and ease of the diseased;" for it was "well known that the surgeons admitted will do no cure to any person but where they know to be rewarded with a greater sum or reward than the cure extendeth unto." † The power of the College of Physicians over the surgeons was decided and arbitrary. The latter were not permitted to administer inward medicines in any disease whatever, and many surgeons were fined and imprisoned for infringing upon the rights of the physicians. In the reign of Elizabeth, two surgeons, Joseph Smart and Edward Messenger, ‡ were each fined five pounds for giving purging potions to patients in surgical cases. In the time of James I. William Foster, a surgeon in Fenchurch-street, was censured for selling a powder for the green-sickness; § and Peter Chamberlayne was fined and imprisoned, notwithstanding the intercession of the Lord Mayor, for selling a few medicines. || During the reign of Charles I. Thomas Cooke, surgeon, for prescribing a few mercurial pills, was obliged to give security by bond of a hundred pounds, "not to practise in the same way of flux-

* 34, 35 Hen. VIII.

† 34, 35 Hen. VIII. In the reign of James II. "Thomas Greenwood, surgeon, was accused by one William Adams, that for the grief of a little skin rubb'd off with his saddle in riding, he promised cure in four days, but physick'd him a fortnight, gave him diet drinks, purged and overheated him, and then sued him for twenty pound for the cure. The censors order'd his imprisonment, and a mulct of five pounds to be inflicted upon him."—Goodall's Account of the Proceedings of the College of Physicians, p. 395.

‡ Goodall's Account of the Proceedings of the College of Physicians, p. 348. § Ibid. p. 363. || Ibid. p. 367.

ing by mercury."* Thomas Bowden was discommuned for giving a purge and diet-drink to a patient for the morbus gallicus; † and Edward Owen was fined, advertised, and imprisoned, for giving mercury, and causing "superpurgation." ‡ Dowton, a surgeon, was fined five pounds, and required to give a bond for two hundred pounds, not to practise midwifery, which he had previously done; § and one Brown, a surgeon, was fined fifty pounds, for *praxis pessima*, giving internal medicines for diseases of the eyes. || The list of surgeons fined and imprisoned by the censors, *propter malam et illicitam praxim et immodestos mores*, is immense, and many were detained until liberated by a *habeas corpus*, although they frequently brought their patients with them to attest the external cures which they had wrought with internal remedies. Some surgeons, indeed, who had been severely handled by the college, disputed their right to interdict the practice of physic by surgeons in surgical cases, and to levy such unmerciful fines and imprisonments. Among these were two men named Jenkins and Read, who having been six several times "fined in small mulcts," and at length imprisoned, procured a writ of *corpus cum causâ* from Sir John Popham, then head chief justice of England, in order to a full hearing of their cause. The chief justice, however, after fully investigating the powers of the college, decided, that in surgical cases, where internal remedies are necessary, a physician was to be called, it being on no such account lawful for the surgeon to invade the physician's province. He confirmed also all the powers of the college, and summed up his opinion by determining that "*no surgeon, as a surgeon, may practise physic, no, not for any disease, though it be the great pox.*"¶ In the year 1595, the College of Physicians sent to the surgeons a friendly letter, to admonish them to abstain wholly from the practice of medicine, which they were compelled to attend to, and which cramped their operations so much, that in a few years afterwards they presented a petition to parliament, to procure an authority for prescribing inward as well as outward medicines in wounds, ulcers, and the French pox. Through the influence of the physicians, however, this petition was rejected, to the no small regret and disappointment of the one party, and the great gratification of the other, who treated the unfortunate petitioners with great resentment and contempt. Such was the abject condition of the surgeons until very recent

---

* Goodall's Account of the Proceedings of the College of Physicians, p. 423. † Ibid. p. 425. ‡ Ibid. p. 333. § Ibid. p. . || Ibid. p. 384. ¶ Ibid. p. 344.

times, times, when by act of parliament they were liberated from the company of barbers, and erected into a royal college, enjoying various privileges and powers independent of the other branches of the profession, but still so far amenable to the rights of the physicians, that at this day it is not decided how far surgeons have a right in any case to interfere with the practice of medicine.

The condition of the apothecaries was not less humiliating than that of the surgeons. The apothecaries and the grocers were formerly one corporation, and tea and rhubarb were bought at the same shop. In the thirteenth year of the reign of James I. the two corporations were separated by law, and the right of preparing and selling medicine was vested solely in the apothecaries' company, and every grocer was restrained from dispensing medicines, by a penalty of five pounds for every month during which he continued the practice. This, however, was the only division of the healing art which the law allowed the apothecaries to perform; they were only the lower limbs of the body medical; the College of Physicians was the sensorium, and the former were to be moved and guided only by their judgment and volitions. They were indeed erected into a company, and endowed with the privileges belonging to similar bodies, such as a power to have a seal, to buy lands, and choose their own officers; but in all matters concerning medicines, they were obliged to consult with the president and four censors of the College of Physicians, who alone could admit members into the company. The physicians were empowered to examine the shops of apothecaries, and to call in the aid of magistrates to enable them to burn and destroy any "evil and fauty stuffe" which they might find therein, and every apothecary resisting such search, was fined ten pounds. They were obliged to deliver to the president and censors the prescriptions of all practitioners who were not members of the college, and were allowed to sell no poisonous drug, unless ordered by some learned physician. The only remedies which it was lawful for apothecaries to prescribe were a few herbs, roots, and waters, and in doing this they only availed themselves of the statute which tolerated the administration of such medicines by old women and magicians, for they were prohibited from the practice of physic, by an act in the third year of Mary. In the reign of Charles I. they infringed so much upon the privileges of the college, that the latter presented a petition to the king, praying that a royal edict might be issued, that under the most severe penalties no apothecary should dare "to compound for the well, well, or administer to the sick, any medicine without the prescription of physicians then living;" and that he that should act contrary should "be punished by the law as a public enemy of the life of man."* The physicians did not fail to exercise the power which the law had placed in their hands, and they used it with so much rigor in those days, that an apothecary could not from his own opinion administer a clyster without danger of fine and imprisonment. In the thirty-seventh year of Elizabeth, "William Chetley, an apothecary, in Bishopsgate-street, having given a lenitive clyster, bolus, and pills, without the advice of a physician, was fined by the censors, and had likewise been committed to prison for his illegal practice, but that some of the fellows interceded for him." † In the reign of James I. Mr. Holland, an apothecary, was fined five pounds for practising medicine, and ordered to be imprisoned without present satisfaction to the college. ‡ George Houghton, an apothecary, was accused of giving pills to a Mr. Robert Roe, counsellor at law, to which his death was attributed; on which occasion the censors declared that it was altogether unlawful for him to give pills, or any other medicine, without the counsel of some approved physician: on a repetition of the same offence, the censors fined him twenty pounds, and committed him to Newgate. § In the same reign, Abraham Hayobert, another apothecary, was for a similar offence fined five pounds, and committed to prison. || In the time of Charles I. one Buggs, an apothecary, was prosecuted by the college, for giving divers medicines and clysters, though he had a degree from the university of Leyden: he was fined fifty pounds, and imprisoned in the Fleet; nor would the Lord Chief Justice grant him a *habeas corpus*, unless with the consent of the College of Physicians, at whose suit he was prosecuted. ¶ In the same reign many apothecaries were summoned before the college, and on being convicted of practising medicine, were fined, discommuned, or imprisoned.

---

* Goodall's Account of the Procedings of the College of Physicians, p. 437. † Ibid. p. 340. ‡ Ibid.

§ Ibid. p. 403. || Ibid. ¶ Ibid. p. 415.

CRITICAL

